# Surgical repair of genital injuries after sexual abuse

**DOI:** 10.3205/iprs000140

**Published:** 2019-09-12

**Authors:** Luz Angela Torres-de la Roche, Harald Krentel, Rajesh Devassy, Maya Sophie de Wilde, Lasse Leicher, Rudy Leon De Wilde

**Affiliations:** 1 University Hospital for Gynecology, Pius Hospital, Carl von Ossietzky University, Oldenburg, Germany; 2 Clinic for Obstetrics and Gynecology. St. Anna Hospital, Herne, Germany; 3 Dubai London Clinic and Specialty Hospital, Dubai, United Arab Emirates

**Keywords:** rape, female genitalia, injuries, gynecologic surgical procedures

## Abstract

**Introduction:** Genital injuries occur in half of cases of sexual assault through digital or penile penetration as well as the use of objects. Women aged >45 years are more likely to have physical injury and anogenital lesions, transmission of STI and HIV. This review focuses on the evidence about surgical reconstruction of the pelvic floor anatomy of adolescents and adult women sexually assaulted during adolescence or adulthood.

**Method:** A systematic literature search was performed in PubMed and Orbis plus for articles published in English and German from June 2008 to June 2018. The literature search was performed in October 2018 by topic combining the following Medical Subject Headings: genital trauma, genital injuries, sexual assault, rape, surgical repair, treatment.

**Results:** 34 records of descriptive studies were identified and 16 full-text articles were included in the present review. Due to the limited number of articles retrieved, articles were not excluded based on methodological design. Superficial genital lesions are common and usually left untreated. For deep vaginal or anal lacerations, intraperitoneal bleeding is usually assessed by means of and additional CT scan or diagnostic colposcopy, cystoscopy, rectoscopy and laparoscopy. Complete reconstruction of the injured is done after. To prevent rectovaginal fistula and uncomplicated primary wound healing a temporary colostomy can be performed.

**Conclusion:** Although most of genital injuries due to sexual assault do not require any major surgical intervention, there is a lack of good quality evidence regarding the best diagnostic and surgical approach to restore deep lesions of genital organs as well lack evidence on contributors to poor wound healing. Therefore, clinical protocols that standardize examination as well as surgical management are encouraged to be developed.

## Zusammenfassung

**Einleitung:** In der Hälfte aller Fälle sexueller Nötigung kommt es durch Penetration mit den Fingern, dem Penis sowie anderen Gegenständen zu Verletzungen im Genitalbereich. Frauen im Alter >45 erleiden häufiger körperliche Verletzungen und anogenitale Läsionen sowie Infektionen mit sexuell übertragbaren Krankheiten und HIV. Schwerpunkt dieser Übersichtsarbeit ist die Evidenz der operativen Rekonstruktion des Beckenbodens von jugendlichen und erwachsenen Frauen, die als Heranwachsende oder Erwachsene Opfer sexueller Gewalt geworden sind.

**Methoden:** Eine systematische Literaturrecherche in PubMed und Orbis plus wurde für den Zeitraum von Juni 2008 bis Juni 2018 durchgeführt und auf die Sprachen Deutsch und Englisch beschränkt. Die Literatursuche wude im Oktober 2018 als thematische Suche mit einer Kombination der folgenden Medical Subject Headings (MeSH) ausgeführt: genital trauma (Genitaltrauma), genital injuries (Verletzungen im Genitalbereich), sexual assault (sexuelle Nötigung), rape (Vergewaltigung), surgical repair (chirurgische Reparatur), treatment (Behandlung).

**Ergebnisse:** Es wurden insgesamt 34 deskriptive Studien identifiziert, wovon 16 Artikelvolltexte in diese Übersichtsarbeit einbezogen worden sind. Angesichts der begrenzten Artikelanzahl wurde das methodische Design der jeweiligen Studien nicht als Ausschlusskriterium berücksichtigt. Oberflächliche Läsionen im Genitalbereich kommen häufig vor und werden meistens nicht behandelt. Im Fall von tiefen Vaginal- bzw. Analverletzungen werden intraperitoneale Blutungen üblicherweise durch CT-Untersuchung oder diagnostische Kolposkopie, Blasenspiegelung, Rektoskopie und/oder Laparoskopie untersucht. Danach werden wiederherstellende Eingriffe des verletzten Bereichs durchgeführt. Um die Bildung von Darmscheidenfisteln und Komplikationen der primären Wundheilung zu vermeiden, kann eine provisorische Kolostomie ausgeführt werden.

**Fazit:** Auch wenn die meisten durch sexuelle Gewalt entstehenden Verletzungen im Genitalbereich keiner größeren chirurgischen Eingriffe benötigen, mangelt es an qualitativ hochwertigen Nachweisen bezüglich einer optimalen chirurgischen und diagnostischen Herangehensweise, um Genitalverletzungen operativ wiederherzustellen. Desweiteren fehlen auch Nachweise über Einflüsse, die zur schlechten Wundheilung beitragen. Um die Untersuchung und das chirurgische Vorgehen zu standardisieren, sollten diesbezüglich klinische Protokolle entwickelt werden.

## Introduction

Sexual assault and sexual gender violence still occur worldwide, mostly against young women and children. At high risk are also persons who are gay, lesbian, bisexual, or transgendered; physically or mentally disabled; homeless, refugees; alcohol and drug users [1]. Physical injury occurs in about half of cases and may include attempted strangulation; blunt traumatic injuries to the head, face, torso, or limbs [2]. Penetrating injuries in the vagina, anus or mouth are made by a penis or other object [1]. However, anogenital lesions are present in one third of survivors and are more likely in those having no prior sexual intercourse (adjusted odds ratio (aOR 4.4, 95% CI 2.4–8.0) [2], [3]. Adolescents usually present with lesions on thighs, labia minora, periurethral area, vaginal vestibule and in the vagina [4]. Women aged >45 years are more likely to have physical injury (OR 2.0, 95% CI 1.2–3.2) and anogenital lesions (OR 2.1, 95% CI 1.4–3.2) [5], and elderly women are at increased risk of vaginal tears and injury, transmission of STI and HIV due to the presence of a thinner and more friable vaginal wall [1].

 It is estimated that one third of survivors never seeks for medical attention and one half only applies 12 hours after the assault [6]. They should receive psychological, medical and legal care by a qualified multidisciplinary team in a comprehensive, respectful and friendly environment [1], [5]. The management should also follow the forensic principles, including maintaining chain of custody of clothes and evidence material, recording of victim’s description of the incident, photo-documentation of physical injuries, elaboration of pictograms, recollection of secretions and blood samples [1], [7]. Referring to police and legal service, is also encouraged [1]. Psychological counselling is recommended to avoid adult sexual re-victimization, future mood disorders such as depression, anxiety, low self-esteem, suicidal tendency and inability to enjoy a healthy sexual life. The medical care includes promptly attention of life threatening and genital injuries, the prevention of sexually transmitted diseases, pregnancy and tetanus [1], [2].

Perineal lesions are graded according to the lesion extension in four grades:

- In the first degree tear, the laceration is limited to the fourchette and superficial perineal skin or vaginal mucosa but the perineal body is intact.
- In the second degree the laceration extends beyond fourchette, perineal skin, and vaginal mucosa to perineal muscles and fascia; but the anal sphincter and the perineal body are not involved.
- In the third degree the fourchette, perineal skin, vaginal mucosa, muscles, and internal- or external anal sphincter are torn.
- In the fourth degree the fourchette, perineal skin, vaginal mucosa, muscles, anal sphincter, and anorectal mucosa are torn [8].

A pre-surgical identification of perineal, anal- and genital lesions is required to determine the medical care that should be provided to the survivor [1], [2]. It allows precise restoration of the injured organs to prevent potentially non-esthetic scars or posttraumatic bladder, bowel and pelvic floor- dysfunction such as urinary urge and stress incontinence, abnormal bladder emptying, fecal incontinence, obstructive bowel disease syndrome and pelvic pain [9], [10]. 

This review focuses on the evidence concerning surgical reconstruction of the pelvic floor anatomy of women sexually assaulted during adolescence or adulthood. The question addressed was: which surgical approaches are recommended to restore the injured genital organs?

## Methods

A systematic literature search was made in PubMed and Orbis plus for articles published in English and German from June 2008 to June 2018. The literature search was performed in October 2018 by topic combining Medical Subject Headings (MeSH): genital trauma, genital injuries, sexual assault, rape, surgical repair, treatment. Observational or controlled studies were, due to the limited number of articles retrieved, not excluded based on methodological design; consequently case reports were also considered for analysis.

## Results

**Figure 1:**
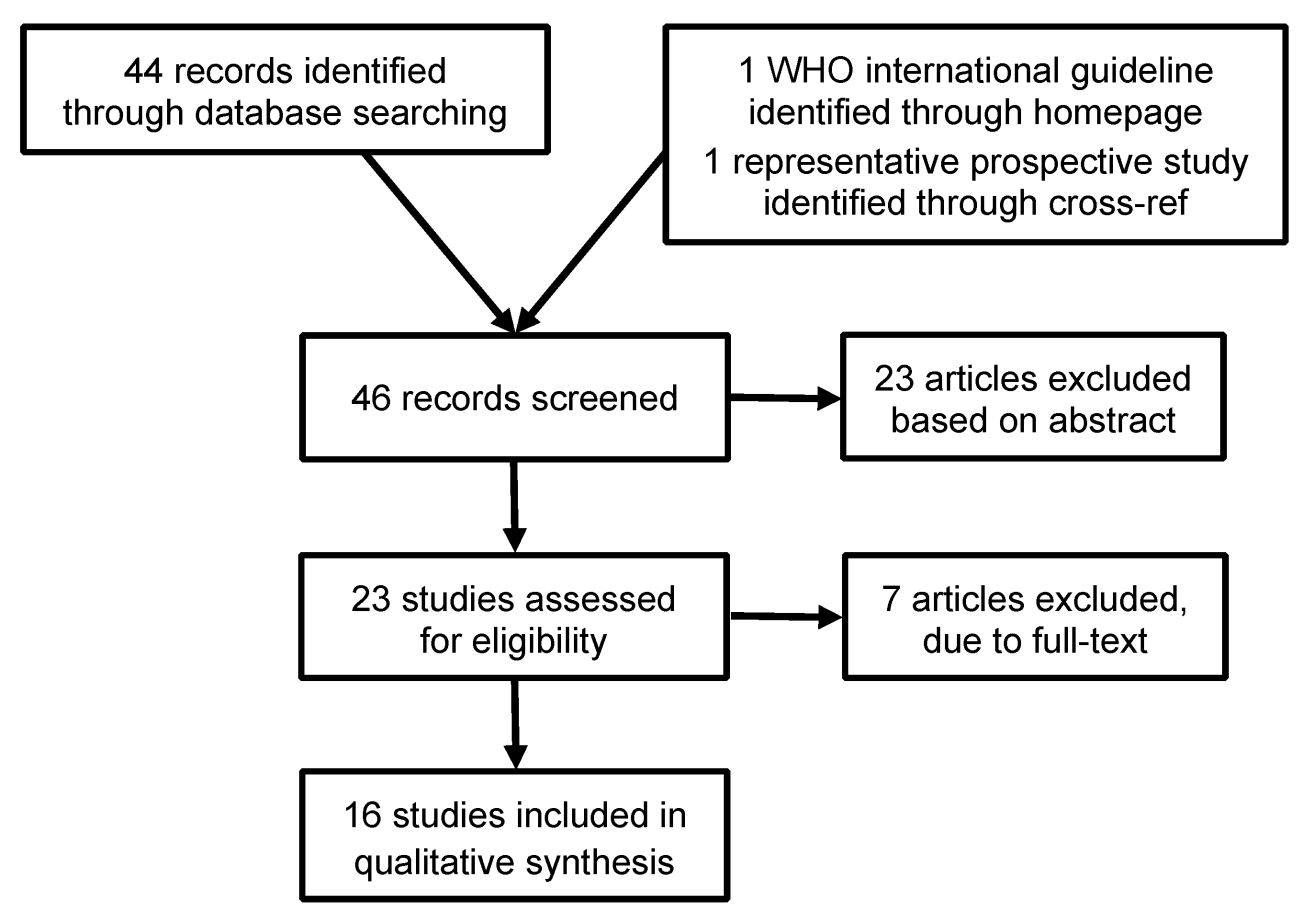
Articles included in the present review

46 records of descriptive studies were identified through database searching. To be included, studies needed to provide a description of the surgical approaches used to repair genital trauma of adolescents or adult sexual assault victims (Figure 1 (Fig. 1)). After abstract screening, 23 articles were excluded because did not report surgical techniques. Of the 16 full-text articles included in the present review, 6 were reviews and 11 case reports. The study populations ranged from 1 to 2.500 participants. The international WHO recommendation paper does not make specific recommendations about the surgical techniques to be used, bat this paper was included as a general guideline about how to identify ani-genital lesions prior to surgical repair.

### Identifying the lesions

According to the WHO recommendations [1], the inspection should be systematically performed. Prior to management, the physician should obtain an informed consent for the procedure, and prepare the required material for an appropriate identification of lesions and for collection of forensic evidence including: “Rape Kit” or supplies for a general and genital physical examination; drugs for STI/HIV prophylaxis; vaccines (Tetanus toxoid, tetanus immuno-globulin, Hepatitis B vaccine); hormonal or Cupper-IUD for emergency contraception; pain relief drugs; and pictograms as well as photo-documentation of body and genitals. If needed, an adequate procedural sedation or anesthesia in the operating room should be offered to perform a complete and accurate genital examination. 

The following order is recommended for genital examination: the mons pubis, inside of the thighs, perineum, anus, labia majora and minora, clitoris, urethra, introitus and hymen. Bruises, scratches, abrasions and tears should be described. If penetration could be occurred, the cervix, the posterior fornix and the vaginal mucosa should be inspected, looking for trauma or bleeding. A bimanual examination and palpation of cervix, uterus and adnexa should be made, looking for signs of abdominal trauma, pregnancy or preexisting infection. If indicated, a rectovaginal examination and inspection of the rectal area for trauma, recto-vaginal tears, preexisting fistulas, bleeding and discharge should be performed. The anal area should be inspected looking for the presence of fecal material on the perianal skin, fissures or rectal tears. Sphincter tone, shape and dilatation of the anus should be evaluated. If there is active bleeding, extended vulvar hematomas, intense pain or suspected presence of a foreign object, supplementary endoscopic examination, colposcopy and vulvo-vaginoscopy should be performed [11]. All features of lesions should be clearly described (Table 1 (Tab. 1)).

### Management of perineal lesions

Conservative treatment by means of nonsteroidal anti-inflammatory drugs and cold packs are recommended for superficial, not extensive vulvar hematomas and for perineal wounds [11], [12]. Compression bandage and rest could be recommended to pain relieve for larger hematomas [11], [12]: the woman is placed on her back and one or more sterile compresses are placed in front of the vulva and her outstretched legs are crossed at about the level of the knee joints. Large and very painful vulvar- and perineal hematomas could require surgical incision with decompression [11], [12]. A urethral catheter may be required to assure the micturition during the healing process [11], [12]. For first and second degree perineal tears, conservative debridement and realignment by primary suturing with resorbable suture under sedation or local anesthesia is usually required [7], [11], [12]. This is done analogous to the treatment of birth perineal injuries [12]. In case of minor vaginal bleeding, a temporary tamponade may help to stop the bleeding [12].

In a prospective study, from 16 children between 4–11 years of age with anogenital lesions after sexual assault, eleven had first and second degree perineal tears like small tears of the hymen, vagina, or the anorectal mucosa, which were managed conservatively [7]. Third and fourth grade lesions were observed in one male and four female between 4–9 years of age, who underwent perineal surgical repair along with the restoration of genital anatomy and diverting colostomy to protect the repair. Approximation of torn vaginal muscles were made with interrupted vicryl 5–0 sutures; transanal repair of mucosal injuries were made from apex to ano-cutaneous junction with vicryl 5–0; repair of the perineal body and sphincter reconstruction were made in layers according to sagittal ano-rectoplasty technique to preserve fecal continence. One girl also required exploratory laparotomy because of vagina vault rupture (colporrhexis). 

Postoperatively, all cases received thorough on-table irrigation of the distal rectum stump, 7–14 days of broad spectrum intravenous antibiotics and alternate-day under sedation examination with pulse irrigations using an antiseptic solution. Warm genital bath, with antiseptic solution, were provided twice daily up to the seventh postsurgical day. Colostomy could be reverted 4–6 months later. Authors reported that all cases healed satisfactory with exception of two cases, one girl developed a rectovaginal fistula three days after surgery that healed without surgical re-intervention and one case had scarred perineum with unsatisfactory cosmetic outcome. 

### Management of the posterior fornix, vaginal vault and penetrating perineal lesions 

Signs of acute abdomen, hematuria, severe vaginal or rectal bleeding have been described in cases of deep laceration of the third vaginal upper, rupture of vaginal walls or penetrating injuries [11]. In the hemodynamically stable patient, a pelvic CT scan to establish the presence of intraabdominal bleeding is usually performed, as well a cysto-and rectoscopy to explore the presence of lesion of urethra and bladder [12]. Primary exploratory laparoscopy or laparotomy are preferred in hemodynamically unstable patients. Debridement and reconstruction of injured organs (bowel, rectum, bladder, and urethra) are promptly performed.

### Management of rectal lacerations

Rectal injuries above the sphincter should be exanimated under anesthesia to asses the extent of the damage and a diverting colostomy can be needed [13]. One study looked at perineal impaling injuries. Authors reported 28 cases of intraperitoneal injuries that were managed by means of colostomy and drainage. In 13 cases of extraperitoneal rectal injuries, distal washouts together with a prospective colostomy as well as drainage were performed with re-anastomosis, leading to satisfactory restitution of organ function. The paper did not mention the time between initial colostomy and subsequent reanastomosis [14].

### Surgical outcomes

Genital and anal injuries requiring surgical care are reported to be low. One study saw 44 females younger than 21 years requiring surgical care for genital or anal trauma out of 11.000 patients which were suspected of having been sexually assaulted. Sexual abuse was reported in 25% of the females, the others were straddle, impalement or motor vehicle accidents [15]. 

Long term outcomes after anogenital trauma were evaluated by means of a colposcope with 35-mm camera attachment in 94 children, 81 of them girls [16]. It was reported that most anal and perineal tears healed without any residual evidence, including labial trauma (n=17), perihymeneal trauma (n=39), hymeneal tears (n=37), and posterior fourchette lesions (n=47). Vascular changes were seen in 2 cases of vaginal tears, 2 cases of perihymeneal trauma and in 4 cases of vaginal lacerations after surgical repair. Anal scarring occurred in 2 of 31 cases [17]. It was also reported that perineal tears grade 1 healed completely while deep injuries grade 3–4 or those that become infected might produce permanent changes. Vulvar hematomas did not distort the patients’ anatomy and usually healed without residual changes, though large hematomas should be treated with incision and drainage as the overlying skin can become necrotic [13]. 

Partial hymeneal tears, abrasions and contusions healed without or with little signs of previous injury within two to fifteen days, depending on the depth and extension of the lesion [18]. The review performed by Berkowitz [17] concluded that superficial laceration to the labia minora, petechiae, abrasions and edema of the hymen healed without residual evidence but deeper and infected injuries or those with repeated disruption could produce permanent changes. Therefore, the presence of permanent changes supports allegations of prior anogenital trauma, but their absence does not exclude the trauma of having been occurred. 

## Discussion

According to the WHO, most sexual assault victims do not seek acute post-rape medical care. Forensic nurse examiners are often the first clinicians to encounter the survivor [1]. When diagnosing the genital trauma, a structured, systemic approach, preferably under sedation or anesthesia, is generally recommended [1]. Psychological support, counseling, evaluation and prophylaxis of possible transmitted infectious diseases and STDs should also be given. Tetanus vaccination should routinely be performed if none within the past 5 years [1]. 

Initial visual evaluation is indispensable. If internal injury cannot be ruled out, image- and minimally invasive diagnosis methods such as CT scan, colposcopy, cystoscopy and rectoscopy are reported to be useful to evaluate the damage extension. Diagnostic laparoscopy or laparotomies are usually performed when intraabdominal hemorrhage, organ perforation or any damage proximal of the anal sphincter is identified [19]. 

Most genital injuries after sexual abuse are reported to be superficial and self limiting, requiring no further evaluation or surgical intervention. Deeper wounds are managed by debridement and primary wound closure to restore structures and organs function [13]. Peritoneal lavage and colostomy after ano-rectoplasty are recommended by some authors to facilitate primary healing process, but the evidence is weak. Primary repair is reported to offer best results in terms of functional as well as cosmetic outcomes while infected injuries or those with repeated disruption are reported to produce permanent changes [19]. Lack of evidence exists about which approach allows best tissue approximation and surgical outcomes. Moreover, there is no published information about possible contributors to poor wound healing, like smoking, chronic disease, concomitant use of medications [20].

Additionally, even the evidence is weak, prophylactic antibiotics are given when puncture, crushing or open wounds were diagnosed [1], [13]. Antibiotics are unlikely to benefit in abrasions and scrapes, however they are mandatory after genital injury with human bites to avoid the risk of aerobic and anaerobic infections [13]. 

Similar to vaginal cuff dehiscence after hysterectomy [21], the management of genital trauma after sexual assault is determined by the clinical circumstances. The surgical approach seems to relay on surgeons experience, availability of resources to assess deep lesions and their intraoperative criteria concerning the closure techniques allowing an adequate tissue approximation and organ restoration.

## Conclusion

There are few published data that focus on management of genital trauma after sexual abuse. Most reports do not give a detailed description of the surgical approach that is used to repair the injured organs, as well as on contributors to poor wound healing. Therefore, protocols that standardize forensic examination as well as surgical management and psychological support are encouraged to be developed in order to offer a comprehensive care.

## Notes

### Competing interests

The authors declare that they have no competing interests.

### Conference presentation

Presentation hold at the 56^th^ annual meeting of the German Society for Interdisciplinary Plastic And Reconstructive Surgery 2018, Ulm, Germany.

## Figures and Tables

**Table 1:**
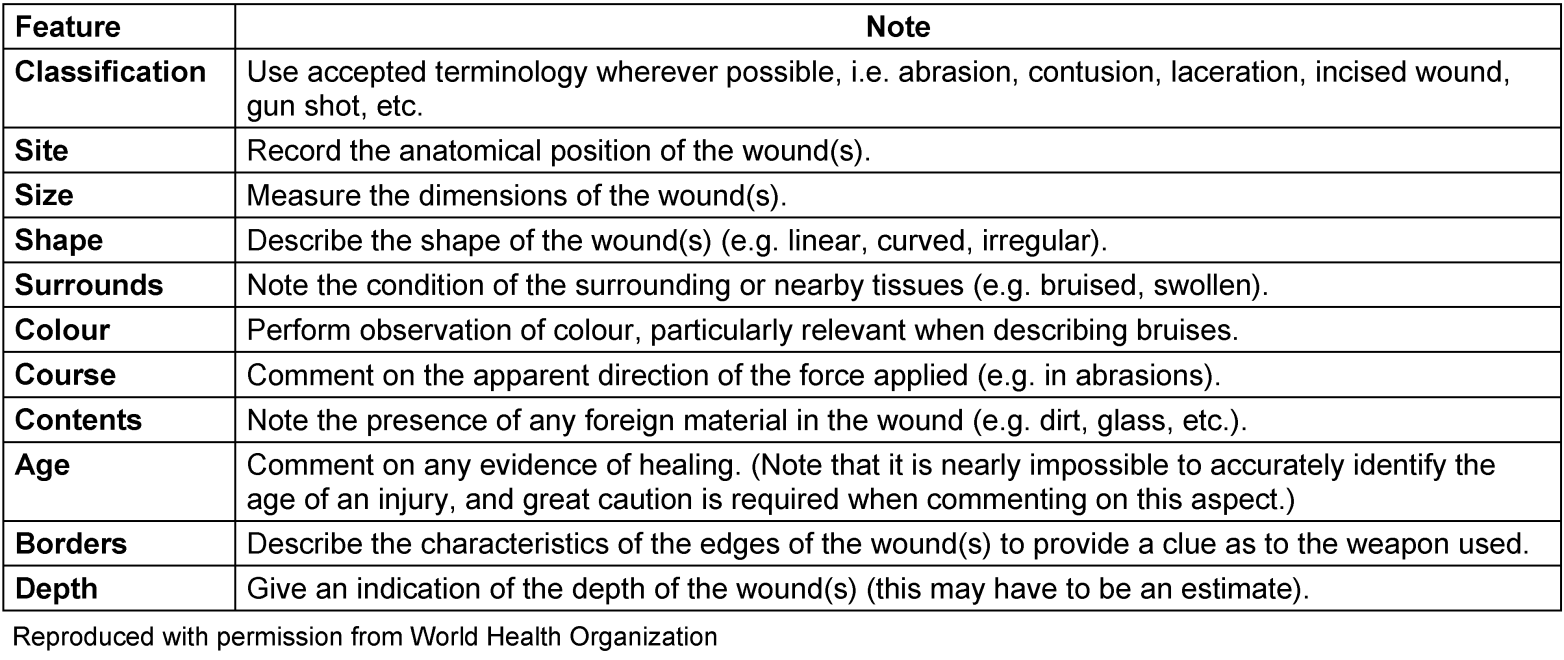
Describing features of physical injuries, according to WHO recommendations [1]
